# Health promotion intervention for people with early‐stage dementia: A quasi‐experimental study

**DOI:** 10.1002/brb3.1888

**Published:** 2020-10-16

**Authors:** Ingelin Testad, Martine Kajander, Martha T. Gjestsen, Ingvild Dalen

**Affiliations:** ^1^ Centre for Age‐related Medicine ‐ SESAM Stavanger University Hospital Stavanger Norway; ^2^ College of Medicine and Health University of Exeter Exeter UK; ^3^ Department of Old Age Psychiatry Institute of Psychiatry, Psychology, & Neuroscience King’s College London London UK; ^4^ Department of Clinical Medicine University of Bergen Bergen Norway; ^5^ Department of Research Section of Biostatistics Stavanger University Hospital Stavanger Norway

**Keywords:** cognition, dementia, depression, education, health promotion

## Abstract

**Introduction:**

With the limited advancements in medical treatment, there is a growing need for supporting people with early‐stage dementia adjust to their diagnosis and improve their quality of life. This study aimed to investigate the effects of a 12‐week health promotion course for people with early‐stage dementia.

**Methods:**

Quasi‐experimental, single group, pretest‐posttest design. A total of 108 persons with dementia participated in this study, and for each participant, a carer was interviewed. The 12‐week health promotion intervention consisted of 2‐hr sessions at weekly intervals. Outcome measures were cognition, measured by Mini‐Mental State Examination, personal, and instrumental activities of daily living (P‐ADL and I‐ADL), measured by Lawton and Brody's Physical Self‐Maintenance Scale and Instrumental Activities of Daily Living Scale, self‐rated health, measured by the European Quality of life Visual Analogue Scale, depressive symptoms, measured by the Cornell Scale for Depression in Dementia, and neuropsychiatric symptoms, measured by The Neuropsychiatric Inventory. Assessments were conducted at baseline and at follow‐up 1–2 months postintervention.

**Results:**

The results demonstrate a small but statistically significant improvement in depressive symptoms (*p* = .015) and in self‐rated health *(p* = .031). The results also demonstrated a small statistically significant decline in the participants’ I‐ADL (*p* = .007). The participants’ cognitive function, P‐ADL, and neuropsychiatric symptoms were stable during the 4‐month follow‐up.

**Conclusion:**

This study demonstrates promising results with regard to the benefit of attending a 12‐week health promotion intervention in promoting health and well‐being in people with early‐stage dementia. With the majority of participants with early‐stage dementia living at home without any healthcare services in a vulnerable stage of the condition, this study makes an important contribution to highlighting the need for, and benefit of, educational approaches for this population.

## Graphical abstract

There are very few services available for people in the early stages of dementia, especially in terms of health promotion. This study explored the effects of a 12‐week health promotion intervention for home‐dwelling people with early‐stage dementia. The findings suggest that a 12‐week health promotion course can improve depressive symptoms and self‐rated health in people with early‐stage dementia. The participants’ cognitive function, P‐ADL, and neuropsychiatric symptoms were stable during the 4‐month follow‐up.

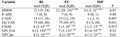

## 1 INTRODUCTION

Worldwide there are more than 50 million people living with dementia. As the world's population grows older, this number is expected to double every 20 years (Patterson, 2018). With the limited advancement in medical treatments, combined with increasing pressures on limited resources, novel and cost‐effective approaches for people with dementia have gained an increased focus (Burgener et al., 2009). Health promotion approaches have the potential to prevent or reduce many avoidable secondary consequences, including injuries and falls, mobility difficulties, nutritional problems, depression, delirium, adverse medication reactions, communication difficulties, or problems performing activities of daily living, all of which can lead to unnecessary hospitalisation and premature nursing home placement (Buettner & Fitzsimmons, 2009). Despite the importance of educating and supporting people with early‐stage dementia, very few services are available for this population. Similarly, research on educational health promotion programs has been very limited. A systematic review (Quinn et al., 2015) found only five studies that reported on structured educational programs for people with dementia and only one of these presented quantitative outcomes.

In this study, we build on a 12‐week health promotion intervention originally developed by Fitzsimmons and Buettner (2003). Health promotion is defined by the Ottawa Charter as “the process of enabling people to increase control over, and to improve their health” (World Health Organization, 1986). Buettner and Fitzsimmons (2009) reported that this intervention resulted in significant positive change in cognition and depression and Richeson et al. (2007) found evidence that health promotion interventions lead to improved self‐efficacy in people with early‐stage dementia. The same intervention was also included in a pilot randomised controlled trial for people with subjective memory problems and showed a significant improvement in cognitive function in the intervention group (Cohen‐Mansfield et al., 2015). Other studies with similar group‐based interventions for people with dementia have also found a positive change in cognition (Laakkonen et al., 2016) and increased self‐efficacy (Quinn et al., 2016).

Overall, these studies provide encouraging evidence that supports educational health promotion programs for people with dementia that attempt to modify lifestyles and habits while the individual is still in the earliest stages of dementia. However, the few studies published lack a robust design, include a homogenous sample and have numerous methodological challenges (Quinn et al., 2015). There is a need for further research to enhance the evidence‐base for health promotion interventions in dementia. Therefore, the aim of this study was to evaluate the effects of attending a 12‐week health promotion course for people with dementia.

## 2 METHODS

### 2.1 Intervention

The format and content of the intervention are based on the health promotion intervention called “Health Promotion for the Mind, Body, and Spirit” developed by Fitzsimmons and Buettner (2003). The intervention has been translated, adapted, and applied to a Norwegian context by researchers at the Centre for Age‐Related Medicine—SESAM, at Stavanger University Hospital.

The intervention consisted of 12 weekly 2‐hr sessions, facilitated by two healthcare professionals. The topics covered at the course are described in Table 1. Each session followed a structured format. First, the session began with a relaxation exercise. Second, handouts were provided. Third, the lead facilitator started discussing that session's topic. Half way through the session the facilitators offered a short break, and refreshments were served. Each session ended with goal setting. During the first session, each participant received a nametag and a booklet. This booklet was a critical component of the educational approach. The carers were not present at the course; however, the participants were encouraged to share the booklet with their carers between sessions (Buettner & Fitzsimmons, 2009). All course facilitators were health care professionals, the majority were nurses and some had previous experience running groups for people with depression or anxiety.

**Table 1:** Topics covered at the 12‐week health promotion course

Session	Title of the session
1	Healthy lifestyles
2	Dementia and delirium
3	Cognitive activities
4	Communication and memory
5	Coping in dementia
6	Physical activity
7	Home and travel safety
8	Recreation & leisure
9	Lifelong learning
10	Medications & talking to your health care provider
11	Nutrition
12	Future planning

The Corbin and Strauss Trajectory Model (Corbin & Strauss, 1991) provided the theoretical basis for the intervention, and it is designed to provide information on the condition process and the development of healthy behaviors in a supportive learning environment to prevent problems that are common in the later stages of the condition.

### 2.2 Study design

This was a quasi‐experimental study, with a single group, pretest‐posttest design. Quantitative assessments were conducted at baseline, that is, prior to attending the 12‐week course, and follow‐up interviews were arranged within 1–2 months after the 12‐week course. Trained research nurses conducted all assessments. The participants were home‐dwelling people with early‐stage dementia, with a carer willing to participate in the study.

Inclusion criteria were as follows: 65 years or older with a diagnosis of early or moderate stage dementia (Clinical Dementia Rating score ≥ 2 (Morris, 1993)), Alzheimer's disease or vascular dementia. In addition, they had capacity to give informed consent, capable of reading and writing, hearing and seeing sufficiently well to work in a group setting and proficient in the language in which the course is provided. Capacity to give informed consent was evaluated by a research nurse informing the person about the study and taking consent, in accordance with local regulations.

Exclusion criteria were as follows: a diagnosis of alcohol abuse, limited life expectancy due to any terminal condition or other serious illness, having ongoing chemotherapy or radiation treatment at enrollment, head injuries, epilepsy, Parkinson's disease, a history of psychiatric illness, a history of a diagnosis of subnormal intelligence and/or prior participation in health promotion or cognitive training programs.

### 2.3 Recruitment and setting

Posters advertising the project were distributed in general practitioners’ offices and local newspapers. Participants were also recruited from the primary healthcare setting, memory clinics, and day care centers. To ensure voluntary participation, healthcare professionals provided contact information to the research team only after ascertaining that those potential participants actually wanted to participate in the study. A research nurse then contacted the participant to review the inclusion criteria and invite them to the study.

The 12‐week health promotion intervention was undertaken in urban and rural cities at five different locations between 2014 and 2019. Those were in the western, eastern, and northern parts of Norway.

### 2.4 Measures

Standard demographic information, that is, age, gender, marital status, and living arrangements, was collected from all participants.

At baseline, level of dementia was assessed with the participant's carer using the Clinical Dementia Rating Scale (CDR) (Morris, 1993) to ensure participants being in the mild or moderate stages of dementia.

Cognition was assessed with the person with dementia using the Mini‐Mental State Examination (MMSE) (Folstein et al., 1975) at both time points. The MMSE is a widely used 30‐point questionnaire, with items assessing orientation, attention, immediate and short‐term recall, language, and the ability to follow simple verbal and written commands. A MMSE score of more than 23 on the Norwegian version indicates minimal or no cognitive impairment (Engedal et al., 1988).

The levels of personal and instrumental functioning were measured by Lawton and Brody's Physical Self‐Maintenance Scale (P‐ADL) and Instrumental Activities of Daily Living Scale (I‐ADL) (Lawton & Brody, 1969) and conducted with the carer. The P‐ADL sum‐score is based on six items (range 0–30), and the I‐ADL is based on eight items (range 0–31), with higher scores indicating a lower function.

Self‐rated health was measured by The European Quality of life Visual Analogue Scale (EQ VAS) (Brooks & EuroQol Group, 1996). The EQ VAS was conducted with the person with dementia and assesses the participants self‐rated health on a vertical, visual analogue scale (VAS), where 0 represents “worst imaginable health state” and 100 represents “best‐ imaginable health state.”

Depressive symptoms were assessed using the Cornell Scale for Depression in Dementia (CSDD) (Alexopoulos et al., 1988). Based on impressions from interviews with both the person with dementia and the carer, the final ratings of the CSDD items represent the rater's clinical impression rather than the responses of the carer or the person with dementia. The scale consists of 19 items that ranges from 0 (absent) to 2 (severe). Total score ranges from 0 to 38, with higher values indicating more depressive symptoms.

Neuropsychiatric symptoms were assessed with the carer using The Neuropsychiatric Inventory (NPI) (Cummings et al., 1994), a 12‐item questionnaire developed to assess behavioral disturbances in people with dementia. NPI is a validated structured interview assessment with a carer. Scores are entered for the frequency and degree of seriousness of each symptom over the last four weeks, and subsequently multiplied into a symptom score. The total possible maximum score is 144. A higher score reflects increased frequency and severity of the disturbances.

### 2.5 Statistical analysis

**Figure 1:**
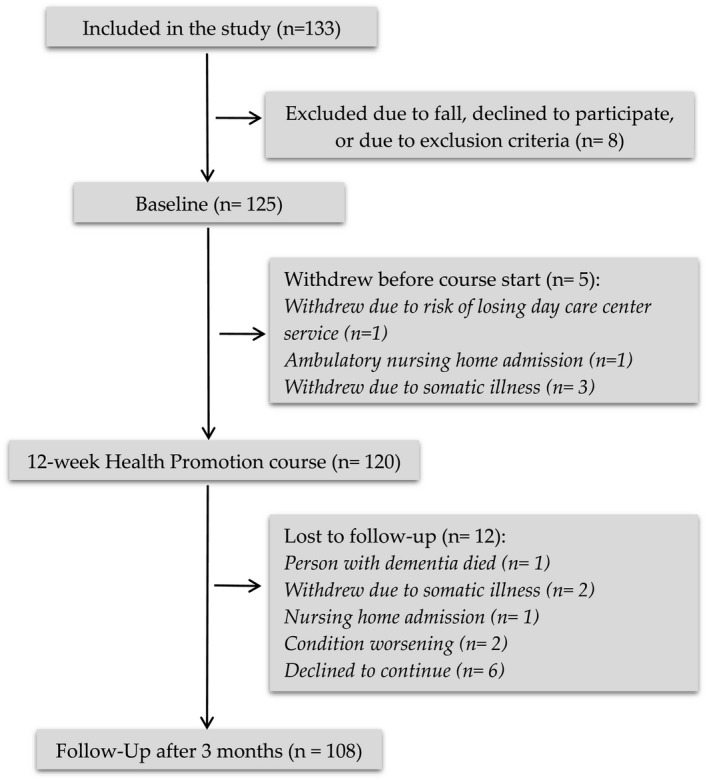
Study flowchart

Participant characteristics were described with count and percentages (categorical variables), or with mean and range (continues variables). All outcome variables apart from the MMSE were either skewed or with outliers, thus were analyzed using nonparametric methods. Outcomes at baseline and follow‐up, as well as changes between baseline and follow‐up, are presented as median and interquartile range (IQR). Changes from baseline to follow‐up were analyzed using the Wilcoxon signed ranks test (paired). Changes in MMSE were also analyzed using parametric methods, from which we present means, standard deviations (*SD*), 95% confidence interval (CI) for change, and p‐value from paired *t* test. Additionally, the observed mean change in MMSE was compared with the expected mean change using a one‐sample *t* test. The IBM SPSS statistical package version 24 for Windows^®^ was used for all statistical analyses. Data from the 108 participants who had completed the course and attended follow‐up were entered into the analysis (see Figure 1). For each outcome variable, only available cases for that outcome variable were included in the analysis. The number of analyzed cases for each outcome variable, if lower than 108, is indicated in the results table (Table 3). For all tests, *p* ≤ .05 was considered statistically significant.

### 2.6 Ethical consideration

The project received formal approval from the Regional Committees for Medical and Health Research Ethics, REC North (2013/2266), and was conducted in accordance with the Helsinki Declaration (World Medical Association, 2013). Written information about the study was sent to all participants before their interviews. At the day of the interview, all participants volunteered written consent after the study procedures had been explained in detail to the participant and their carer. The trial protocol is registered with ClinicalTrials.gov, Identifier: NCT03741543.

## 3 RESULTS

Table 2 depicts demographic details of the participants and their carers. Overall, the majority of the participants had higher education, lived with their spouse or domestic partner and did not receive any healthcare services. It was a requirement that participant attended all sessions, which was the case in most of the groups. However, there were a few of the participants that could not be present at all sessions, but everyone was present minimum 9 of 12 sessions. There were some dropouts at follow‐up (Figure 1).

**Table 2:** Baseline characteristics of people with dementia and their carer

Participant characteristics	Person with dementia (*n* = 108)	Carer (*n* = 108)
Gender		
Male	52 (48%)	40 (37%)
Female	56 (52%)	68 (63%)
Age, mean (range)	77 (65–89)	65 (39–86)
Participant's marital status		
Married	68 (63%)	
Widowed	26 (24%)	
Divorced/separated	10 (9%)	
Domestic partner	1 (1%)	
Single/never married	3 (3%)	
Living arrangements		
Living with relative/partner/friend	69 (64%)	
Living alone	39 (36%)	
Level of education		
Primary (10 years education)	31 (29%)	
Secondary (13 years education)	22 (20%)	
College/university	53 (49%)	
Other	2 (2%)	
Health services		
None	64 (59%)	
Home‐based care	13 (12%)	
Day care center	13 (12%)	
Home help	1 (1%)	
Activity friends	1 (1%)	
Home help and day care center	10 (9%)	
Home nursing and home help	5 (5%)	
Home help and day care center	1 (1%)	
Clinical dementia rating scale score		
0.5 very mild dementia	56 (52%)	
1 mild dementia	45 (42%)	
2 moderate dementia	7 (6%)	
Carer's relationship to person with dementia		
Spouse/domestic partner		65 (60%)
Daughter/son/grandchild		38 (35%)
Niece/Nephew		1 (1%)
Friend		3 (3%)
Other		1 (1%)

Note

Descriptive statistics presented as count and percentages unless specified.

### 3.1 Outcomes

Table 3.

### 3.2 Effects of the intervention on cognition

Table 3 shows no change in cognition of people with dementia according to MMSE during the 4‐month follow‐up, using the Wilcoxon signed ranks test (*p* = .95). Parametric analysis of MMSE also demonstrated a stable MMSE, with a mean baseline score of 21.9 (*SD* 3.4) and mean follow‐up score of 22.0 (*SD* 3.5), a mean difference of 0.0 (*SD* 2.8; 95% CI −0.5 to 0.5), *p* = .97 (paired samples *t* test). Expected decline in MMSE for people with Alzheimer's disease during one year is 3.2 (Rongve et al., 2016) or 3.3 (Breitve et al., 2014). Based on this, we can assume that the expected change during 4 months is approximately −1. Our observed change in mean MMSE is statistically significantly different from an expected change of −1 (*p* < .001, one‐sample *t* test).

**Table 3:** Changes from baseline to follow‐up 1–2 months postintervention, *n* = 108

Variable	BL	FU	Diff	
	med (IQR)	med (IQR)	med (IQR)	*p*
MMSE	22 (19, 24)	22 (20, 24)*^n^* ^ = 106^	0 (−2, 2)*^n^* ^ = 106^	.95
P‐ADL	7 (6, 8)	7 (6, 8)	0 (0, 1)	.40
I‐ADL	15 (11, 18)	15 (12, 19)	1 (−1, 3)	.007
EQ VAS	75 (60, 80)	75 (69, 87)	0 (−5, 10)	.031
CSDD	4 (2, 6)*^n^* ^ = 106^	3 (1, 6)*^n^* ^ = 106^	−1 (−2, 1)*^n^* ^ = 105^	.015
NPI_FxS	6 (3, 14)*^n^* ^ = 105^	7 (3, 13)*^n^* ^ = 99^	0 (−4, 5)*^n^* ^ = 96^	.32
NPI_dis	4 (2, 9)*^n^* ^ = 102^	4 (1, 9)*^n^* ^ = 96^	0 (−3, 2)*^n^* ^ = 91^	.65

Note

Descriptives given as median (interquartile range; IQR). MMSE: Mini‐Mental State Examination (0–30), P‐ADL: Physical Self‐Maintenance Scale (0–30), I‐ADL: Instrumental Activities of Daily Living Scale (0–31), EQ VAS: Self‐rated health (0–100), CSDD: Cornell Scale for Depression in Dementia (0–38), NPI_FxS: The Neuropsychiatric Inventory frequency × severity (0–144), NPI_dis: The Neuropsychiatric Inventory occupational disruptiveness (0–60), BL: Baseline, FU: Follow‐up, Diff: Changes from baseline to follow‐up.

### 3.3 Effects of the intervention on ADL

The median change in I‐ADL was one point, which demonstrated a statistically significant decline in the participants’ instrumental functioning (*p* = .007). No significant effects of the intervention were observed on P‐ADL, with stable scores during the 4‐month follow‐up.

### 3.4 Effects of the intervention on self‐rated health

Wilcoxon signed ranks test demonstrated statistically significant change in EQ VAS between baseline and follow‐up (*p* = .031). The median change was zero, but the IQR’s demonstrate a transition toward higher EQ VAS values (Table 3).

### 3.5 Effects of the intervention on depression

Depression measured by CSDD demonstrated a significant median decline by one point (*p* = .015), that is, a statistically significant improvement in depressive symptoms.

### 3.6 Effects of the intervention on neuropsychiatric symptoms

Neuropsychiatric inventory scores were based on frequency multiplied with severity and occupational disruptiveness. None of these changed significantly between baseline and follow‐up, with median differences of zero, and *p*‐values .32 and .65, respectively.

## 4 DISCUSSION

The aim of this study was to evaluate the effects of home‐dwelling people with early‐stage dementia attending a 12‐week health promotion course. In line with previous research, this study demonstrated a significant improvement in depressive symptoms in people with dementia. Depressive symptoms are common in people with dementia and are associated with reduced quality of life (Winter et al., 2011) and functional decline (Starkstein et al., 2005). The majority of participants in this study did not receive any healthcare services. People with early‐stage dementia are in a vulnerable stage of the condition. With limited follow‐up after diagnosis and with very few available services, many people are facing the condition alone and have many unanswered questions, which can lead to depression and challenges adjusting to and coping with the condition (Gorska et al., 2018; The Norwegian Government, 2015). At the 12‐week course, the participants receive information and support, and learn about coping skills to manage the condition and related symptoms, as well as meeting others in the same situation. Our findings support Buettner and Fitzimmons’ (2009) finding that attending a 12‐week health promotion course can significantly reduce depressive symptoms in people with dementia. In contrast to Buettner and Fitzsimmons' (2009) study, we included a substantially larger intervention group with a more diverse sample of people from both urban and rural areas. Furthermore, our study replicated the finding using a different depression scale, the Cornell scale. The Cornell scale is considered the most accurate assessment of depressive symptoms in people with dementia, because it combines interviews with both the participant and their carer (Enache et al., 2011).

Another encouraging finding was in the area of self‐rated health. After attending the health promotion course, participants had a significant improvement in self‐rated health. The course focuses on health promotion and provides information on the importance of staying healthy and active while living with a chronic condition. The findings are consistent with the feedback from both participants and carers at follow‐up interviews, where they reported feeling more motivated and encouraged to participate in health promoting activities. Results from similar group‐based interventions have been conflicting. In Quinn et al.’s (2016) study, participants attending a self‐management group rated themselves as having a lower health‐related quality of life at 3‐month assessment, but improved at 6‐month follow‐up. A study conducted by Laakkonen et al. (2016) evaluating a self‐management intervention for people with dementia and their spouses found no change between intervention and control group regarding health‐related quality of life. Although the amount of change in our study was modest, Buettner and Fitzsimmons (2009) also demonstrated a significant change in health habits in intervention group participants. Improvements in health‐related behaviors have the potential to increase overall functioning in people with dementia and contribute to prevent conditions that are common in the later stages of the condition (National Alzheimer's Association, 2007).

The cognitive function of people with dementia was stable during the 4‐month follow‐up and the result is statistically significantly different from the anticipated average decline in AD (Breitve et al., 2014; Rongve et al., 2016). Our results are in line with previous research. Buettner and Fitzsimmons (2009) found a significant improvement in the intervention group's MMSE scores, whereas the control group significantly declined. There might be a learning effect with tests spaced 4 months apart. However, in the study of Buettner and Fitzsimmons (2009) the control group, which was assessed at the same time points as the participants in our study, had a 1.18 point mean decline in MMSE. Similarly, Laakkonen et al. (2016) found that participants in the intervention group's cognitive scores improved more than those of the control group, assessed by clock drawing test and verbal fluency test. Through the 12‐week course, the participants socialised and challenged their brains, which is found to be especially beneficial for people with dementia, as it can help preserve cognitive skills (Woods et al., 2012).

### 4.1 Strengths and limitations

In comparison with previous intervention studies (Buettner & Fitzsimmons, 2009; Laakkonen et al., 2016; Quinn et al., 2016), we recruited a substantially larger sample size (108 persons with dementia and 108 carers), maintained high levels of follow‐up, and included a more diverse sample including participants from both urban and rural regions. The lack of control group is a limitation in this study and should be taken into consideration when interpreting the results. Without a control group, there is a high degree of uncertainty linked to the findings, because a variety of factors taking place in the participants’ lives or changes within the participants can have caused the changes between baseline and follow‐up and not necessarily the intervention (Polit & Beck, 2017). The outcome measures used in this study could also be a limitation. According to Buettner and Fitzsimmons (2009) and Gaugler et al. (2011), the standard measures used in dementia studies may not be sensitive enough for people in early‐stage dementia, and thus, not sensitive enough to capture the specific benefits of this intervention. The measures’ lack of sensitivity could be the reason for the small changes observed between baseline and follow‐up in this study.

## 5 CONCLUSION

This 12‐week health promotion course demonstrated promising results in promoting health and well‐being in people with early‐stage dementia. With the majority of participants with early‐stage dementia living at home without any healthcare services in a vulnerable stage of the condition, this study makes an important contribution in highlighting the need for, and benefit of, educational approaches for this population. More research on the effect of health promotion interventions for people with early‐stage dementia with a control group design is needed. Furthermore, future research also needs to explore the qualitative experiences of people with dementia attending this type of course.

## CONFLICT OF INTEREST

None declared.

## AUTHOR CONTRIBUTIONS

IT involved in study concept, study recruitment, data collection, oversight of data analysis, interpretation of findings, and writing the manuscript. MK involved in development of the study, study recruitment, data collection, interpretation of findings, and writing the manuscript. MTG involved in development of the study, setting up the study, study recruitment, data collection, and critical review of manuscript. ID involved in development of the study, statistical data analyses, interpretation of findings, and critical review of manuscript. All authors have read and approved the manuscript.

### Peer Review

The peer review history for this article is available at https://publons.com/publon/10.1002/brb3.1888.

## Data Availability

The data that support the findings of this study are available from the corresponding author upon reasonable request.
